# Akt and c-Myc Induce Stem-Cell Markers in Mature Primary p53^−/−^ Astrocytes and Render These Cells Gliomagenic in the Brain of Immunocompetent Mice

**DOI:** 10.1371/journal.pone.0056691

**Published:** 2013-02-12

**Authors:** Josefine Radke, Ginette Bortolussi, Axel Pagenstecher

**Affiliations:** Department of Neuropathology, Philipps University of Marburg, Marburg, Germany; University of Michigan School of Medicine, United States of America

## Abstract

Astrocytomas and their most malignant variant glioblastoma multiforme (GBM) represent the vast majority of primary brain tumors. Despite the current progress in neurosurgery, radiation therapy and chemotherapy, most astrocytomas remain fatal disorders. Although brain tumor biology is a matter of intense research, the cell-of-origin and the complete astrocytoma-inducing signaling pathway remain unknown. To further identify the mechanisms leading to gliomagenesis, we transduced primary astrocytes on a p53^−/−^ background with c-Myc, constitutively active myr-Akt or both, myr-Akt and c-Myc. Transduced astrocytes showed oncogene-specific alterations of morphology, proliferation and differentiation. Following prolonged periods of cultivation, oncogene-transduced astrocytes expressed several stem-cell markers. Furthermore, astrocytes coexpressing c-Myc and Akt were tumorigenic when implanted into the brain of immunocompetent C57BL/6 mice. Our results reveal that the loss of p53 combined with oncogene overexpression in mature astrocytes simulates pivotal features of glioma pathogenesis, providing a good model for assessing the development of secondary glioblastomas.

## Introduction

Astrocytoma and glioblastoma represent the majority of primary tumors of the central nervous system [1]. Even well differentiated tumors bear a grim prognosis for the patients due to the diffuse infiltration of the surrounding brain that prevents complete resection of the glioma [2]. Low grade diffuse astrocytomas show an almost complete propensity to progress to malignant anaplastic astrocytoma and subsequently to glioblastoma. Median survival of the latter is about one year [3]. Despite considerable efforts to characterize the cell-of-origin leading to glioma, it remains unknown whether these tumors are derived from a mature, differentiated astrocyte or a central nervous system (CNS)-progenitor cell [4]–[6]. Two subtypes of glioblastoma have been characterized: primary and secondary glioblastoma. Both types, although histologically largely indistinguishable, differ significantly with regard to histogenesis and common genetic alterations. Primary glioblastoma develop *de novo* without a proof of a preceding less malignant astrocytoma. By contrast, secondary glioblastomas evolve by malignant progression of low-grade or anaplastic astrocytomas. The majority of secondary glioblastomas bear mutations of the genes encoding the tumor protein 53 (p53) and of isocitrate-dehydrogenase 1 (IDH-1), leading to the perception that these mutations are early, if not the earliest, steps in the development of these gliomas [7], [8]. Indeed, impaired function of p53, either due to mutation of *TP53* or due to genetic alterations that interfere with proper function of p53 such as murine double minute oncogene (*MDM2*) amplification, is a common observation in astrocytic gliomas and glioblastoma [9].

In order to understand the initiation of glioma formation, a large number of different murine and human glioma models have been developed. Malignant transformation of astrocytes deficient for p53 occurs after prolonged passaging and can be modulated by specific culturing conditions [10], [11]. Combined deficiency for p53 with mutated neurofibromin 1 (NF1) led to various gliomas in transgenic mice underlining the role p53 in gliomagenesis [12]. The effect of oncogenes such as c-Myc or Akt on gliomagenesis was investigated in the context of a combination with Ras expression [13]–[16]. This approach, however, had the drawback that in contrast to several other cancers, Ras mutations are rarely detected in malignant gliomas [17]. Combined deletion of p53 and phosphatase and tensin homolog (PTEN) in the CNS of mice induced gliomas that showed upregulated c-Myc [18]. Inaddition, inactivation of PTEN that is often seen in human glioblastoma leads to activation of the Akt pathway. The necessity to combine these defects is explained by the fact that functional p53 counteracts the deleterious effects of c-Myc in several ways. The ARF/p53 pathway is activated by unphysiologically high levels of c-Myc which leads to apoptosis [19], G_2_ arrest of fibroblasts [20] and represses c-Myc [21]. To further elucidate and mimic the features of human astrocytoma, we developed a mouse model employing mature primary astrocytes deficient for p53 that overexpress c-Myc, myristoylated (myr), i.e. constitutively active Akt or both c-Myc and myr-Akt, respectively. Here, we demonstrate that primary p53^−/−^ astrocytes that coexpress c-Myc and Akt develop a progenitor-like differentiation and are tumorigenic when implanted into the brain of C57BL/6 mice. In contrast to many transplanted tumor cell lines these tumors are not immunogenic. The tumorigenic nature of these cells combined with their stem cell-like phenotype makes them a valuable tool for further investigations of gliomagenesis.

## Materials and Methods

### Primary astrocyte cell culture

Mice heterozygous for the p53 mutation on a C57BL/6 background were bred in order to obtain littermates with the three genotypes p53 ^+/+^, p53^+/−^ and p53^−/−^ [11]. Primary astrocytes were isolated from one day old p53 ^+/−^ and ^−/−^ p53^+/+^ mice as described previously with the exception of minor modifications [22]. Briefly, the brain was removed from the skull and the meninges were discarded. Subsequently, the cortices were minced and the cells were dissociated by trituration through a 2 ml pipet and followed by incubation with trypsin (Invitrogen, Carlsbad, USA). The cells were seeded in 10 cm plates in DMEM (Invitrogen) containing 10% FCS (PAA, Linz, Austria) and 1% penicillin/streptomycin (Invitrogen). The genotype of the cells with regard to p53 was confirmed by PCR from tail DNA of the respective animal. After two culture passages the majority of cells (>95%) were astrocytes, as verified by immunoreactivity for glial fibrillary acidic protein (GFAP).

### Retroviral transduction

Phoenix-ECO cells [23] were transfected with pBabe-Puromycin-MycIIaWT, pWZL-Neo-Myr-Flag-AKT1[24] and pBabe-H2B-GFP (all vectors were a generous gift of M. Eilers, Würzburg, Germany), respectively, according to standard protocols [23]. Retroviral supernatant was gently removed 48 and 66 hours after transfection, filtered through a 0,45 µm filter and stored at −80 °C pending transduction of primary astrocytes. Transduction was done according to standard protocols [23] with the exception of minor modifications. Successful transduction was determined in an Enhanced Green Fluorescent Protein (EGFP)-transduced control culture and by antibiotic selection (puromycin/gentamycin). The EGFP-transduced p53^−/−^ astrocyte cultures were followed up to exclude a transduction-induced phenotype. Six independent sets of p53^−/−^ astrocyte cell cultures were stably transduced with c-Myc (p53MYC), Akt (p53AKT) or c-Myc/Akt (p53MA). Transduced cultures were compared to non-transduced p53^−/−^ astrocyte cell cultures (p53Ctrl) that served as control cultures. Astrocyte cultures that were transduced with Akt, c-Myc or both, Akt and c-Myc were kept in culture for up to six months post transduction.

### Immunocytochemistry

Briefly, astrocytes were grown on cover slips (Ø 10 mm, Menzel-Gläser, Braunschweig, Germany), washed in PBS (Invitrogen) and fixed in methanol/acetone (1:1) at −20 °C for 2 min and stored at −20 °C until staining. Fixed cells were blocked in 0.01 g/ml bovine serum albumin (Sigma-Aldrich, Munich, Germany), 0.01% Triton X-100 (Sigma-Aldrich), 0.01% NaN_3_ in PBS) for 30 min or in 100 µl of 5% goat serum for 30 min. Subsequently, astrocytes were incubated with the following primary antibodies overnight at 4°C: Rabbit anti-GFAP (Dako Glostrup, Denmark), rat anti-mouse Ki67 (Dako), rabbit anti-CD133 (Abcam, Cambridge, England), rabbit anti-nestin (Lifespan Bioscience, Seattle, USA), rabbit anti-Musashi-1 (Millipore, Darmstadt, Germany) and rabbit anti-Olig2 (Millipore). After thorough washing, cells were incubated with appropriate secondary antibodies conjugated to Alexa dyes (Invitrogen), or to horseradish peroxidase and subsequently stained with DAB (Vector Labs, Burlingame, USA). Nuclei were counterstained with 4′,6-Diamidino-2-phenylindol (Dako) and hematoxylin, respectively. Omission of the primary antibody resulted in no staining.

### Histology and immunohistochemistry

Histology and immunhistochemistry were performed on 5 µm-thick cryostat sections. Hematoxylin and eosin stains were prepared according to standard procedures. For immunohistochemistry, sections were blocked with 5% goat serum for 30 min and incubated with the following primary antibodies over night at 4°C: Rat anti-CD4 and anti-CD8 (both from BD-PharMingen, San Jose, USA) and rat-anti Mac-1 (generous gift from M. Simon, MPI Freiburg, Germany). Sections were washed and incubated with a biotinylated goat anti rat IgG (Dako). Staining was done according to the manufacturer's protocol of Histostain® Plus Kit (Invitrogen). Omission of the primary antibody resulted in no staining.

### Proliferative index

For each cell line and time point, the number of Ki67-immunoreactive (IR) nuclei in a total of 50 DAPI-labeled nuclei was counted. For each condition, 7 fields of at least three individual experiments were counted. The percentage of proliferating cells was calculated as the ratio of Ki67-IR nuclei to DAPI-labeled nuclei multiplied by 100.

### Cell count

Control and transfected astrocytes were grown to confluence in 10 cm culture dishes. At each passage suspended astrocytes were counted using a hemocytometer. The number of astrocytes that would have resulted if all cells had been passaged was computed as the total astrocyte number at a certain passage multiplied by the product of the dilution factor(s) of previous passage(s). The results of three independent experiments are represented as mean ± SD.

### Stereotaxic implantation of astrocytes

Cells were harvested, washed, and resuspended in DPBS at a final concentration of 10^4^ cells/µl and kept on ice until intracerebral injection. Mice on a C57BL/6 background were anesthetized with isoflurane (Baxter, Deerfield, USA) and placed in a stereotaxic unit. Cells were injected over a period of 5 minutes into the right striate body (2.0 mm lateral, 0.6 mm anterior to the bregma and 3.3 mm below the skull surface). After 21 days the mice were sacrificed, the brain was dissected, and cryostat sections were prepared. The sections were either stained with hematoxylin and eosin or used for immunohistochemistry as described above. Tumor volume was calculated by measuring the diameter and length of each tumor in the respective slide with the maximum tumor size [25]. Approximation for a rotational ellipsoid (spheroid) was performed with the following equation: V = 4/3 π (D/2)^2^ L/2, with V =  Volume, D = diameter and L = length. One superficial tumor had the shape of a triangle, for this tumor the size was calculated with the formula for a cone: V = 1/3 π (D/2)^2^ H, with H = height of the triangle. This study was carried out in strict accordance with the German legislation on animal welfare. The protocol was approved by the local governmental committee on animal welfare (Regierungspräsidium Giessen, permit Number 74-2010).

### RNA Isolation, probe set and RNase protection assay (RPA)

Astrocytes were rinsed with DPBS, homogenized in Trizol® (Invitrogen) and total RNA was extracted according to the manufacturer's protocol. For every condition total RNA of three independent transduction sets was isolated for each investigated culture passage and analyzed with a probe set described before [26]. To determine the expression of human c-Myc in transduced astrocytes by RPA a specific 236 bp long target sequences was used to generate a probe against c-myc (NM_002764.4; nucleotides 1404 to 1639) as described previously [22]. RPA was performed as described before using 2.5 µg of total RNA [27]. Autoradiographs were scanned and quantitatively analyzed by the ImageJ (1.42q) program (National Institutes of Health, Bethesda, USA).

### Western blot

Astrocytes were washed with PBS, trypsinized and harvested by centrifugation. Immunoblotting was performed as described before with minor modifications [28]. Briefly, cell pellets were homogenised on ice in 100 µl Tris-EDTA buffer (pH 7.6) containing 1% Nonidet P-40, 5 µg/ml Pepstatin A and a protease and phosphatase inhibitor cocktail (Complete™, PhosSTOP™, Roche). Protein concentration was determined using Bradford's reagent. The protein lysates were stored at −80 °C pending immunoblotting. 50 µg of each protein lysate were electrophoretically fractionated on 10% Tris/glycine gels at 200 V. Following electrophoresis, the samples were transferred to a PVDF membrane (Immobilon, Millipore, Bedford, MA) and blocked with 5% skim milk powder in TBS. The membranes were incubated with polyclonal antibodies against rabbit anti-CD133, rabbit anti-nestin, rabbit anti-Musashi-1 and rabbit anti-Olig2 (same antibodies as given above). A subsequent incubation with an antibody against GAPDH (Chemicon, Temecula, CA) was performed to determine equal protein loading of each lane. Band density was determined with the NIH Image 1.62 software and each value was normalized to the band density of the respective GAPDH band.

### Statistical analysis

Data of the proliferation index and RNA-expression were analyzed by one-way analysis-of-variance (ANOVA) and Tukey's test using GraphPad Prism 4.00 software (GraphPad Software, San Diego, CA). For the analysis of cell counts log-regression, curves were compared using a linear mixed-effects model fit and a simultaneous test for general linear hypotheses was performed to adjust the p-values for multiple testing with the statistical software R (www.r-project.org), version 2.15.0 and multiple packages. Differences with a p value < 0.05 were considered to be statistically significant.

## Results

### Expression of c-Myc, Akt or both oncogenes alters the morphology of astrocytes

To determine the influence of active c-Myc and Akt either alone or in combination, p53^−/−^ and p53^+/+^ astrocytes were purified from neonatal mouse cortex, propagated for about 14 days and plated at a density of 4×10^6^ cells per 10 cm dish. After 24 hours, astrocytes were transduced with either EGFP, c-Myc, Akt or both oncogenes. Control transductions performed with EGFP revealed a much lower transduction efficiency in p53^+/+^ astrocytes compared with p53^−/−^ astrocytes. The p53^−/−^ astrocytes tolerated transduction with the oncogenes without a significant loss of cells while transduction of p53^+/+^ astrocytes with c-Myc or Akt was not successful as most cells died shortly after transduction. Therefore, in our hands, p53 deficiency was essential for successful transduction of murine astrocytes with c-Myc and Akt. The growth characteristics and cytological phenotype of transduced cultures were compared to non-transduced p53^−/−^ (p53Ctrl) and p53^+/+^ astrocyte cultures. The p53^+/+^ astrocytes exhibited the typical star-shape (Figure S1A) and a strong immunoreactivity to GFAP (data not shown). The p53^+/+^ astrocytes demonstrated a steady rate of growth and maintained a stable saturation density for three passages after which they showed an increased time to reach confluence. Most cells died after approximately 6 weeks. Early-passage p53Ctrl cultures were undistinguishable from p53^+/+^ astrocytes (Figure S1B). However, single p53Ctrl astrocytes showed either an increased size of the nucleus or they were multinucleated (Figure S1B). With continued passaging p53Ctrl astrocytes developed an increased nuclear-cytoplasmic ratio as well as increased proliferation. After two to three months (7 passages), many cells died. Some cultures of p53Ctrl astrocytes survived for more than six months, however, due to low cell numbers these cells were not in the subject of further experiments. Most early-passage p53AKT astrocytes exhibited an astrocytic shape. Many cells, however, were multinucleated and showed an increased nuclear size and prominent nucleoli (Figure S1C). With continued passaging, an increasing number of p53AKT astrocytes exhibited an increased size and were frequently multinucleate with enlarged pleomorphic nuclei and prominent nucleoli (Figure S1 F, I). Cell death, indicated by rounded bright cells that detached from the surface of the dish, was observed only occasionally in p53AKT cultures during the six month observation period. The p53MYC astrocytes showed very early distinct morphological alterations. The cells were spindle shaped and had an increased nuclear-cytoplasmic ratio (Figure S1 D). At later passages, a high number of dead cells were observed in p53MYC cultures (Figures S1 D and K) and after three to four months the majority of these cells died. The morphology of astrocytes expressing both, c-Myc and Akt (p53MA) was very similar to astrocytes expressing c-Myc only, however, there were few cells that resembled the very large multinucleated cells that were observed in astrocytes that expressed Akt only (Figure S1 E). In sharp contrast to p53MYC cell cultures, p53MA cells revealed only few dead cells during the six months of observation demonstrating the protective effect of active Akt (Figures S1 H and L).

### Differentiation of transduced astrocytes

**Figure 1:**
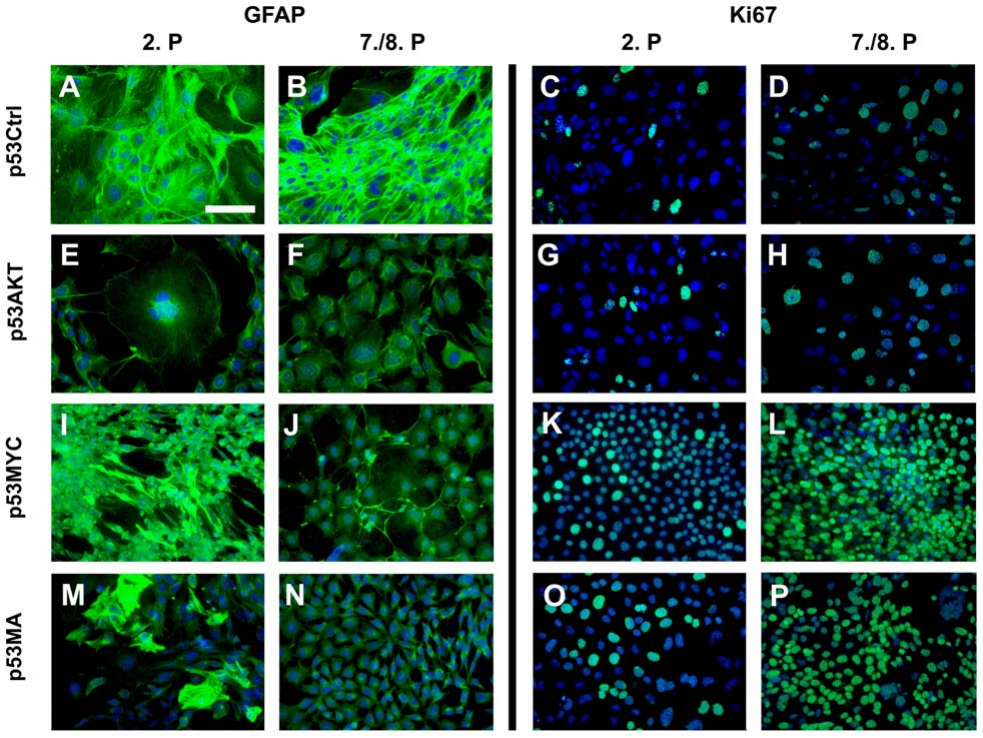
Phenotype of astrocytes expressing constitutively active Akt and/or c-Myc. The p53Ctrl astrocytes of the 2^nd^ (A) and the 7^th^ (B) passage revealed strong GFAP-immunoreactivity (-IR). In p53AKT astrocytes the GFAP-IR was significantly downregulated as early as the 2^nd^ passage and stayed low through the (E) 8^th^ passage (F). The p53MYC astrocytes showed strong GFAP-IR in the 2^nd^ passage (I) while there was only low GFAP-IR in the 8^th^ passage (J). Astrocytes expressing Akt and c-Myc showed scattered cells with and without GFAP-IR in the 2^nd^ passage (M) and virtually complete loss of GFAP-IR at later passages (8^th^ passage, N). The number of Ki67-IR cells was low in the 2^nd^ passage (C) and slightly increased in the 8^th^ passage (D).The Ki67-IR of p53AKT astrocytes was comparable to p53Ctrl astrocytes at both time points (2^nd^ (G); 8^th^ (H) passage, respectively). Expression of c-Myc immediately induced a strong increase in the Ki67-IR (2^nd^ passage, K) that further increased to the 8^th^ passage (L). The p53MA astrocytes showed a high number of Ki67-IR nuclei (2^nd^: O; 8^th^: P) in both passages. (Scale bar: 50 µm)

In order to determine the astrocytic differention of transduced astrocytes we analzyed the expression of glial fibrillary acidic protein (GFAP) by immunocytochemistry and by RNase Protection Assay (RPA). Initially >95% of cells were GFAP-immunoreactive astrocytes. Wildtype astrocytes from p53^+/+^ and astrocytes from p53^−/−^ cultures showed no difference in GFAP-immunoreactivity (data not shown). To further determine the astrocytic differentiation of cultured astrocytes in the course of progressive passaging, we determined the expression of GFAP. Cells from the 2^nd^ (Figure 1A) as well as the 7^th^ (Figure 1B) passage of p53Ctrl astrocytes showed strong GFAP-immunoreactivity (IR). In contrast to this, Akt expression led to a dramatic loss of IR for GFAP as early as the second passage (2^nd^ passage: Figure 1E; 8^th^ passage: Figure 1F). Astrocytes expressing c-Myc showed preserved IR for GFAP at the 2^nd^ passage (Figure 1I) which was lost in most cells at the 8^th^ passage (Figure 1J). Most astrocytes that expressed both c-Myc and Akt showed a significant loss of GFAP-IR at the 2^nd^ passage (Figure 1M) which decreased even more at later stages (Figure 1N).

**Figure 2:**
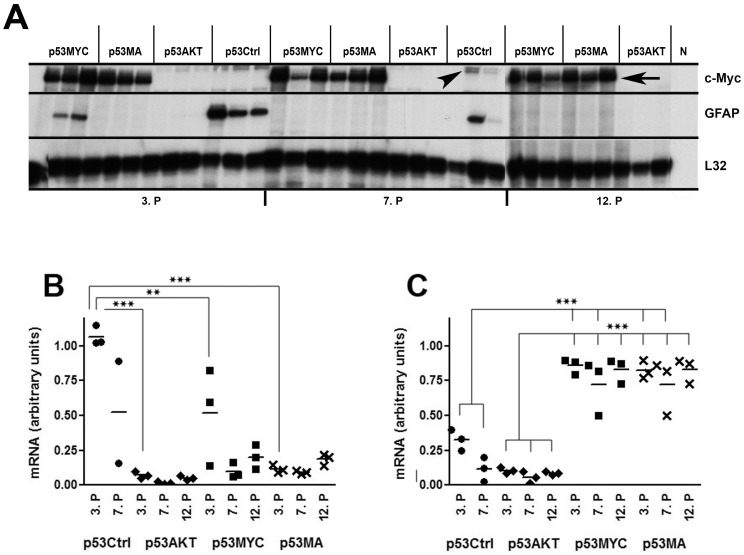
GFAP and c-Myc gene expression in astrocytes. A: RPA was performed with 2.5 µg of total RNA of three independent astrocyte cultures for each condition. Constitutively expressed ribosomal Protein L32 RNA was used as a loading control. N: Negative control (tRNA). Autoradiographs were scanned and quantitatively analyzed using the ImageJ (1.42q) program. Strong c-Myc-specific mRNA expression (A, arrow) was observed in p53MA and p53MYC cultures (A, C). The RPA probe for c-myc revealed a weak constitutive band (A, arrowhead) in the RNA from cells that were not transduced for c-Myc (p53Ctrl and p53AKT). GFAP-specific mRNA was detected in p53Ctrl (because of partial degradation of the RNA one sample could not be quantitatively analyzed) and early-passage (3^rd^) p53MYC (A, C). p53AKT and p53MA showed no detectable amounts of GFAP-specific mRNA in all passages tested (A,B). ** p≤0.01, *** p≤0.001.

The RPA results corroborated these findings. The expression of GFAP and c-Myc mRNAs were determined for transduced astrocytes at the 3^rd^, 7^th^ and 12^th^ passage. Control astrocytes (p53Ctrl) showed strong expression of the GFAP-RNA at the 3^rd^ and 7^th^ passages (we never were able to generate a 12^th^ passage of control astrocytes because p53Ctrl cells died after about six weeks of cell culture) while expression of c-Myc led to slightly lower expression of GFAP-RNA at the 3^rd^ passage and repressed GFAP-RNA expression under the detection limit at passages 7 and 12 (Figures 2 A, B). Expression of GFAP-RNA was eliminated by Akt at all passages tested.

The RPA probe for c-myc revealed a weak constitutive band in the RNA from cells that were not transduced for c-Myc (p53Ctrl and p53AKT, Figure 2A, arrowhead) that migrated at a slightly higher molecular weight than the band for c-Myc that was expressed at high levels in astrocytes transfected with c-Myc (p53MYC and p53MA, arrow, Figure 2 A, C). In order to determine the purity of the primary astrocyte cultures and to investigate whether the expression of c-Myc and/or Akt induced the expression of neural differentiation markers we performed RPA with a probe set containing neuronal (GAP43, NF68, synaptophysin, calbindin), astrocytic (GFAP) and oligodendroglial (PLP, MBP) genes. This analysis revealed that the primary astrocytes contained no significant amount of contaminating neuronal or oligodendroglial cells. Moreover, this analysis demonstrated that transduction of primary astrocytes with AKT and/or c-Myc did not induce the expression of neuronal or oligodendroglial genes (data not shown).

### Proliferation kinetics of transduced astrocytes

**Figure 3:**
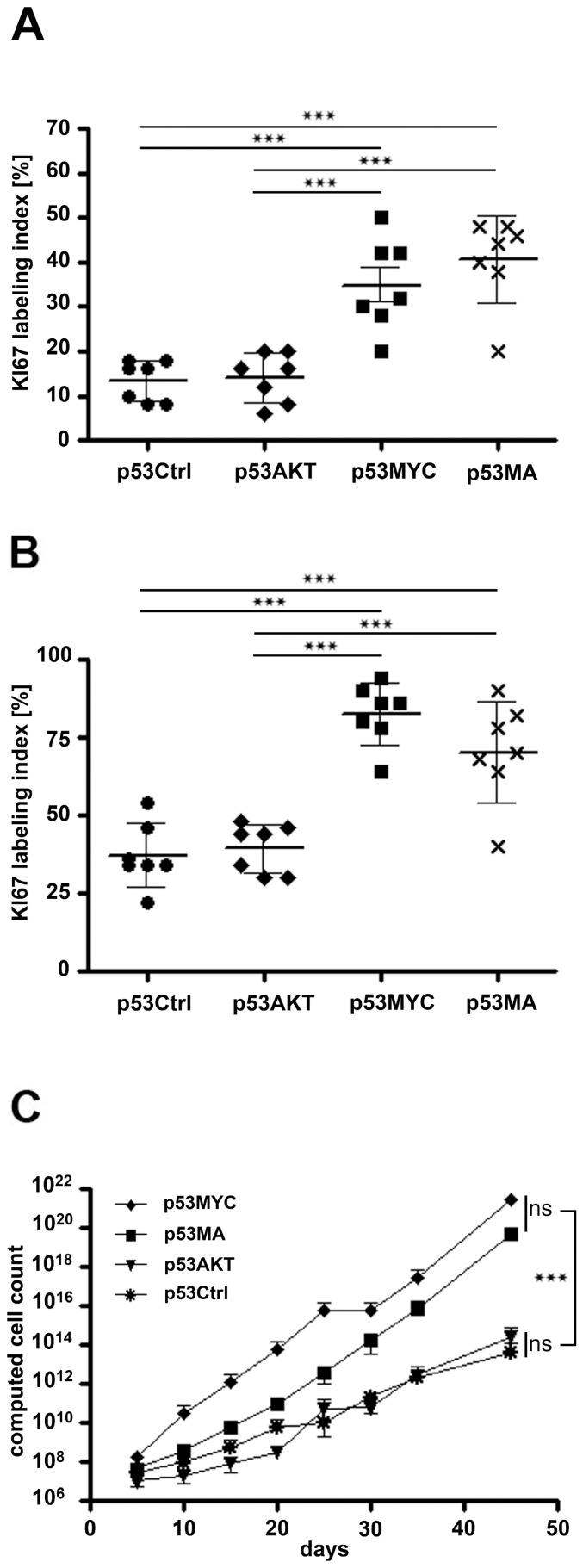
Proliferation kinetics of control and oncogene transduced astrocytes. The Ki67-LI of the four cell lines at **A**: the 2^nd^ passage and B: the 7^th^ passage. C: Cells of each cell line were counted at every passage for a period of 45 days. The theoretically derived cell number was computed for each cell line. The p53MYC and p53MA astrocytes showed high proliferation. The p53Ctrl and p53AKT revealed a much lower and comparable growth rate. The mean±SD of three independent experiments is shown. _***_ p≤0.001.

The proliferation kinetics of control and transduced astrocyte cultures were determined by immunocytochemical staining for the proliferation-associated antigen Ki67 at different passages and by counting the cell number over a prolonged period of 45 days. Primary wildtype C57BL/6 astrocytes showed very low numbers of Ki67-IR nuclei (data not shown). By contrast, primary p53^−/−^ control astrocytes showed an increased ratio of of Ki-67-IR to DAPIlabeled nuclei (Ki67-labeling index (Ki67-LI)). At the 2^nd^ passage p53Ctrl astrocytes (Figure 1C) had a similar Ki-67-LI as Akt expressing astrocytes (p53AKT) (Figure 1G). In strong contrast to this, p53MYC and p53MA astrocytes of a 2^nd^ passage demonstrated a significantly higher Ki-67-LI (Figures. 1K and 1O). At the 7^th^/8^th^ passage, p53Ctrl (Figure 1D) and p53AKT (Figure 1H) still showed virtually identical Ki67-LIs, however, at this time point, a larger growth fraction was observed in both cell lines. Again, 8^th^ passage p53MYC and p53MA astrocytes (Figures 1L and 1P) revealed a higher Ki67-LI, which in p53MYC astrocytes reached up to 90% while p53MA cells had a slightly lower growth fraction. Quantification of these findings revealed statistically significant differences between the Ki67-LI of p53Ctrl and p53AKT astrocytes on the one hand and p53MYC and p53MA cells on the other hand (Figure 3 A and B). These results were further corroborated by the proliferation kinetics of the different astrocyte lines as determined by counting cell numbers at every passage of representative transduced and control cultures over a period of 45 days. The cell counts of three representative transduction sets were used to calculate the number of theoretically derived astrocytes of each cell line after 45 days (Figure 3C). In line with the comparable Ki-67-LI p53Ctrl and p53AKT astrocytes showed similar growth characteristics (Mean total cell number was 10^13^ for p53Ctrl, 10^14^ for p53AKT, statistically no significant difference (p = 0.934)), while p53MA astrocytes proliferated to mean of 10^19^ cells and p53MYC to 10^21^ cells (statistically significant difference between p53Ctrl and p53AKT on one side and p53MYC and p53MA on the other; p≤0.001, no difference between p53MYC and p53MA; p = 0.922; Figure 3C).

### c-Myc and Akt induce stem cell markers in primary p53^−/−^ astrocytes

**Figure 4:**
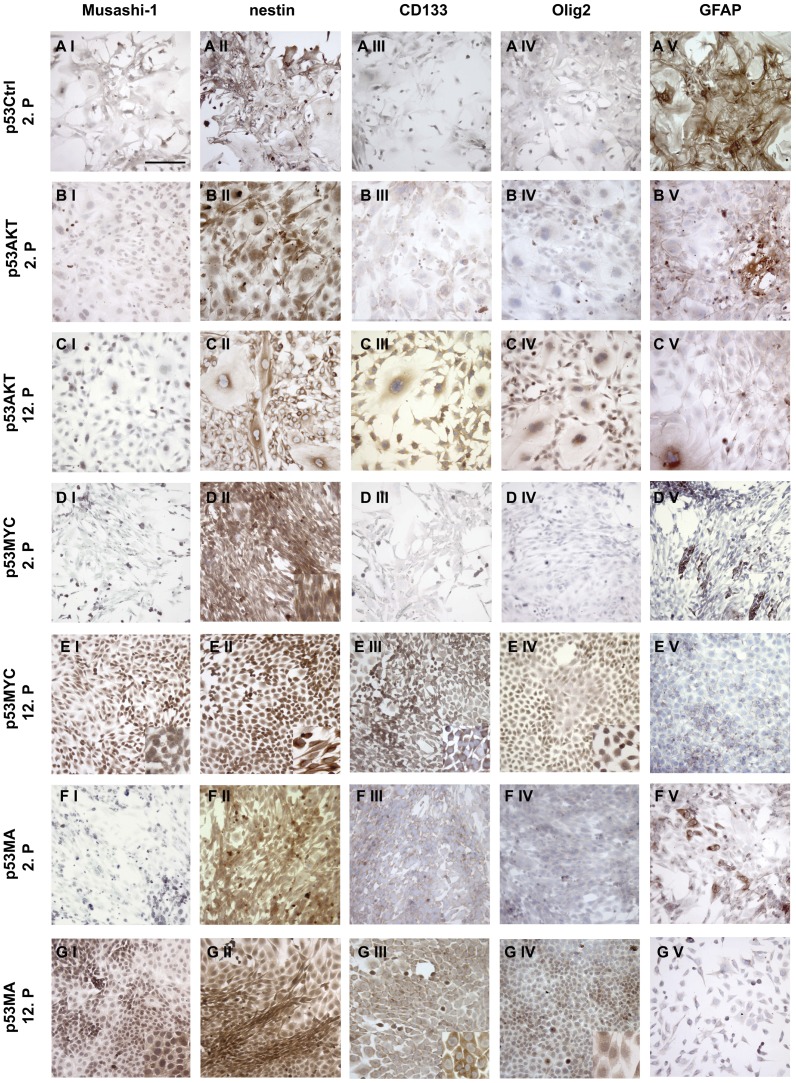
Expression of c-Myc and Akt induces stem cell marker expression in primary p53^−/−^ astrocytes. Control cultures (p53Crtl) showed no stem cell marker expression (A I–A IV) and strong GFAP-IR (A V). All oncogene-transduced astrocyte cultures showed a significantly decreased GFAP-IR in early passages (2^nd^) (B V, D V, F V) and complete loss of GFAP-IR in late (12^th^) passages (C V, E V, G V) and induced expression of nestin (B II–G II). At late time points (12^th^ passage) expression of Akt additionally induced CD133 (C IV) and Olig2 (CV). Expression of c-Myc led to immunopositive for Musashi-1, nestin, CD133 and Olig2 (E I–IV, G I–IV) and negative for GFAP (E V, G V) (Scale bar: 100 µm).

**Figure 5:**
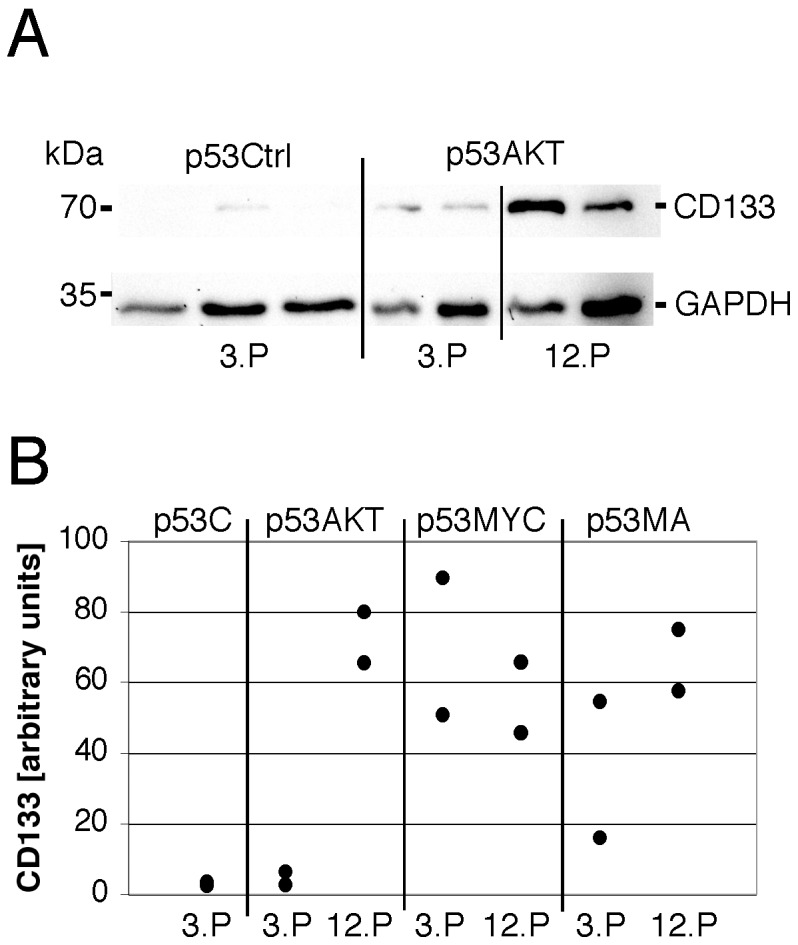
Upregulation of CD133 in transduced astrocytes. A: Immunoblots for CD133 and GAPDH of protein extracts of control cultures (p53Ctrl) and Akt transduced astrocytes (p53AKT) of the 3^rd^ or 12^th^ passage are shown. B: Normalized band density of CD133 of control cultures (p53C), and transduced astrocytes of the 3^rd^ or 12^th^ passage. In p53AKT and p53MA there is a strong upregulation of CD133 between the 3^rd^ and the 12^th^ passage.

Recently, it has been proposed that only certain cells in glioblastomas have the potential for tumor initiation. These cells have been labeled glioblastoma stem cells (GSCs) or tumor initiating cells (TICs) to emphasize their capacity for self-renewal, proliferation and differentiation. In order to determine whether the expression of Akt, c-Myc or both oncogenes in primary p53^−/−^ astrocytes may induce the development of stem cell features, we performed immunocytochemistry for the stem cell markers Musashi-1, nestin, CD133 and Olig2 on control and oncogene-transduced astrocytes at different time points after transduction (Figure 4). Control p53^−/−^ astrocytes of a 2^nd^ and a 7^th^ passage revealed strong GFAP-IR and were negative for Musashi-1, nestin, CD133 and Olig2 (Figure 4 AI-AV). Both, c-Myc as well as Akt either alone or in combination with each other induced nestin-IR from the early passage 2 on (Figure 4 B II-G II). At the 12^th^ passage, additional induction of CD133 and Olig2 expression was detected in astrocytes transduced with Akt, c-Myc or both oncogenes (Figure 4 CII-CIV, EII–IV, GII–IV). Moreover, astrocytes expressing c-Myc or both (Ak/c-Myc) were immunoreactive for Musashi-1 at the 12^th^ passage (Figure 4 EI, GI). The upregulation of CD133 in transduced astrocytes was also demonstrated using protein extracts of the cells from 3^rd^ and 12^th^ passages (Figure 5). While there was no or only low level expression of CD133 in 3^rd^ passage control p53^−/−^ astrocytes, transduced astrocytes showed a strong upregulation of CD133 in particular at later passages. The majority of CD133 was detected as an immunoreactive band that migrated at 70kDa corresponding to a truncated form of CD133 [29]. Together, these results indicate that primary cortical astrocytes are able to develop a cytologically de-differentiated phenotype resembling that of GSCs.

### Astrocytes expressing c-Myc and Akt are tumorigenic in the brain and are tolerated by the immune system

**Figure 6:**
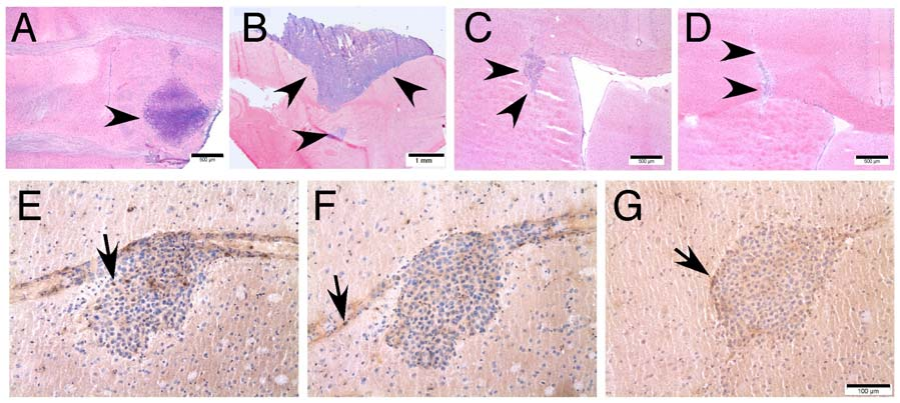
Tumorigenic potential of p53MA astrocytes. Injection of p53MA astrocytes of a 3^rd^ passage resulted in the development of tumors (arrows) of similar size in C57BL/6 (A) and Rag 2^−/−^ (B) mice, while p53MA astrocytes of an 8^th^ passage led to clearly larger tumors (C). Tumors in C57BL/6 mice revealed only few CD4+ (D, arrow)-, no CD8+ (E) - T-cells and no MAC-1 immunoreactive macrophages (F, scale bar: A–C: 500 µm, D–F: 100 µm).

**Figure 7:**
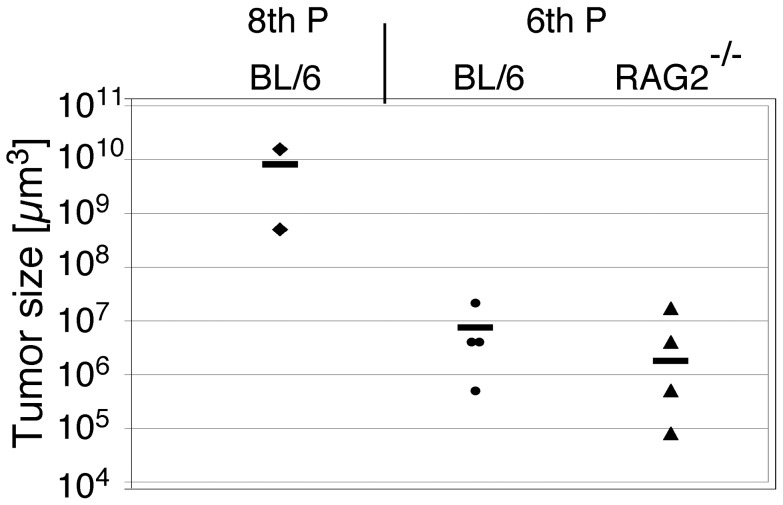
Tumorsize in wild type and immunocompromized mice. While 8×10^4^ p53MA cells of an 8^th^ passage produced very large tumors in mice on a C57BL/6 (BL/6) background, 6×10^4^ p53MA astrocytes induced considerably smaller tumors. The comparable size of the tumors in BL/6 and RAG2^−/−^ mice indicated that there was no immune response against the tumor cells in immunocompetent C57BL/6 mice. Tumor volume was measured as described in Materials and Methods.

To determine the tumorigenic potential of astrocytes expressing both c-Myc and Akt, we injected p53MA astrocytes stereotactically into the striate body of C57BL/6 mice. We used cells of early passages for these experiments in order to implant slowly proliferating cells with a limited number of accumulated mutations. Mice were sacrificed 21 days after implantation of the astrocytes, the brains were dissected, cryostat sections prepared and tumor formation evaluated. Two out of three C57BL/6 mice implanted with 8×10^4^ astrocytes of an 8^th^ passage developed very large tumors (0.5 mm^3^ and 15.6 mm^3^), one of which extended to the surface of the brain (Figure 6 A and B, 7, Table 1). The larger tumor showed areas of graphic necrosis and numerous mitotic figures and demonstrated a sharp border towards the surrounding brain. This tumor resembled the tumors that establish from glioma cell lines such as GL261 cells when grafted into the mouse brain. In order to establish infiltrating tumors, lower cell numbers of an earlier passage were injected into the striate body. All mice (n = 4) injected with 6×10^4^ p53MA astrocytes of a 3^rd^ passage developed small tumors (Figure 6, 7 and Table 1). Since the survival and proliferation of transferred astrocytes might be compromised by invading immune cells, we implanted 6×10^4^ p53MA astrocytes of a 3^rd^ passage into the striate body of C57BL/6 Rag2^−/−^ mice that are devoid of functional lymphocytes. After 21 days, all mice (n = 4) showed small solid tumors in the brain. The size of these tumors was comparable with the tumors that developed in wildtype C57BL/6 mice (Figures. 6 and 7). These slowly developing tumors revealed a smoother edge towards the surrounding brain, however, an infiltrating behaviour comparable to the human disease was not observed. Immunohistochemical stainings were performed to identify infiltrating T-cell subtypes and macrophages. In wild type C57BL/6 mice, there were only occasionally CD4+ T-cells and neither CD8+ T-cells nor MAC-1 immunoreactive macrophages were detected in the transplants (Figure 5 D-F). The similar size of tumors in wild type and Rag2^−/−^ mice together with the absence of invading mononuclear cells in and around the transplants in wild type mice indicates that there was no significant immune response against p53MA astrocytes in the brain of congenic C57BL/6 mice.

**Table 1:** Tumorigenicity of p53^−/−^ astrocytes expressing c-Myc and Akt in the brain of C57BL/6 and Rag2^−/−^ mice.

recipient mouse	C57BL/6	C57BL/6–Rag2^−/−^	C57BL/6
# passage/# cells	3^rd^/6 _*_ 10^4^	3^rd^/6 _*_ 10^4^	8^th^/8 _*_ 10^4^
tumors developed/mice implanted	4/4	4/4	2/3

## Discussion

### Mature primary cortical astrocytes as glioma initiating cells

Our understanding and the therapy of most gliomas still is in the fledgling stages. Although the pathways that lead to gliomas have been extensively studied [7], [30], the cell of glioma origin still has not been unequivocally resolved [31]. Moreover, we are far from a cure for the vast majority of primary brain tumors. Mutations of the p53 tumor suppressor gene are a common finding in low grade astrocytomas and glioblastomas, and patients with a germline mutation of p53 (Li-Fraumeni-syndrome) are predisposed to develop brain tumors and other malignancies at an early age [32]. The loss of function of this tumor suppressor is one of the early and essential steps in the development of astrocytomas and secondary glioblastoma [33]. Mice deficient for p53 show increased proliferation and early acquisition of further genomic alterations in subventricular zone (SVZ) stem- and progenitor cells [34]. These findings have been underlined by a more recent report that demonstrated that conditional deletion of the p53 binding domain (p53^?E5–6^) in GFAP-expressing cells, i.e. radial glia and mature astrocytes, leads to GBM-like tumors in mice after 6 months of age [35]. The earliest-stage tumor cells were detected in the immediate vicinity of the SVZ and the rostral migratory stream (RMS) indicating that SVZ-cells are the glioma initiating cells in this mouse model. Lineage marker analysis suggested that mutant SVZ-B stem cells generated SVZ-C-like glioma precursors [35]. Importantly, in the mouse models for p53 dependent gliomagenesis mentioned above mature cortical astrocytes were not found to give rise to tumors. On the one hand this finding underlines the similarity to human astrocytoma and glioblastoma that very often develop in the periventricular regions. On the other hand, it has been shown that in the adult human cortex there is considerable proliferation of astrocytes [36]. A recent report demonstrated that in the neonatal mouse cortex, most astrocytes are generated by proliferation of cortical astrocytes [37]. These data suggest that mature astrocytes have the potential for tumor formation as soon as these cells acquire mutations of crucial genes such as IDH-1 or p53. This idea is supported by *in vitro* data showing the higher growth fraction, better survival, significant genetic instability and tumorigenic potential of p53^−/−^ astrocytes [11], [38], [39]. With increasing number of passages, p53^−/−^ astrocytes show a strong potential to form subcutaneous and intracerebral tumors in nude mice [10]. Human gliomas show malfunction of several pathways such as p53-MDM2-p14ARF, RB1-p16INK4a, PTEN/Akt-1, IDH-1/2 or EGFR-dependent signaling [40]–[42]. Glioma models can exploit these pathways in order to emulate certain features of the human disease. Virally transduced expression of active Ras and Akt induces glioma formation from nestin-expressing neural progenitors but not from GFAP-expressing astrocytes in mice [13]. Additional expression of c-Myc, however, renders GFAP-expressing astrocytes tumorigenic [14]. In this system, omission of Ras, that is combined expression of c-Myc and Akt only, in GFAP-expressing cells, led to only one tumor in 27 mice. In later studies, it was shown that p53 and Pten concomitantly act on c-Myc in the control of differentiation and self-renewal of stem cells in glioma [18], [43]. In the present study, we therefore focused on the effects of combined deficiency for p53 and expression of c-Myc and/or Akt. In order to elucidate whether the early steps of gliomagenesis can be recapitulated by starting from mature primary astrocytes, we transduced p53^−/−^ cortical astrocytes with active c-Myc and/or Akt, oncogenes that are implied in the development of human glioblastoma [18], [44]. C57BL/6 wild type astrocytes showed massive cell death after transduction with c-Myc. This result is consistent with the reported counteraction of p53 on the signaling pathways of both c-Myc as well as Akt. Non-physiologically high levels of activated c-Myc induce p53-dependent apoptosis [45] and G_2_ arrest in fibroblasts [20]. Moreover, c-Myc expression is repressed by p53 via miRNA [21]. Deficiency for p53 was therefore essential for a successful expression of c-Myc and Akt in our astrocyte cultures. *In vitro* c-Myc drives rapid growth, cell cycle progression, senescence and promotes de-differentiation [46]. In our experiments, astrocytes on a p53^−/−^ background that were transduced with c-Myc showed a significant increase in proliferative and mitotic activity and a distinct change of their morphology towards a de-differentiated, bipolar phenotype. Moreover, p53MYC astrocytes showed a loss of GFAP expression and concomitant strong expression of nestin, suggesting the development of an undifferentiated neural precursor cell phenotype. p53MYC astrocytes additionally expressed CD133, Olig2 and Musashi-1 which are considered to be neural stem cell (NSC) markers observed in undifferentiated brain tumor initiating cells (BTIC) [47]. After prolonged culturing periods of four months, we observed massive cell death of c-Myc transduced astrocytes which might be due to p53-independent suppression of Bcl-2 and induction of pro-apoptotic proteins like Bax and Bak [48]. By contrast, astrocyte cultures transduced with Akt (p53AKT and p53MA) were protected from cell death within the 6 month observation period. Akt is a potent inhibitor of Bad and Caspase 9 [49] and therefore mediates cell survival and inhibition of apoptosis. Our findings correlate to human glioblastoma where the mutation of PTEN is one of the major genetic alterations which results in an increased Akt activity in the tumor cells [50]. Together, the combined overexpression of both, c-Myc and Akt in p53^−/−^ astrocytes modeled several features of gliomagenesis.

### Expression of stem cell markers by transduced mature p53^−/−^ astrocytes

As outlined above, the CSC or TIC has yet to be unequivocally determined [31]. Gliomas may arise from transformed neural stem or progenitor cells and GFAP-expressing astrocytes, respectively [30], [51]. These cells show similarities with *sensu stricto* stem cells such as proliferation, self-renewal and multi-potency [52], [53]. Several proteins such as CD133, nestin, Olig2 and Musashi-1 are expressed by neuronal precursor cells and are considered markers for GSCs or TICs [47]. In the model presented here, early-passage primary p53Ctrl astrocytes presented with a mature astrocytic phenotype (GFAP^+^, nestin^−^, Olig2^−^, Musashi-1^−^, CD133^−^). This expression pattern was maintained throughout extended passaging (7 passages, 60 days). Transduction with Akt and to a lesser degree with c-Myc led to an early downregulation of GFAP-expression. These findings agree with previously reported changes in astrocyte differentiation by c-Myc and Akt [14], [54]. Neither c-Myc nor Akt induced a neuronal or oligodendroglial differentiation in primary astrocytes as assessed by the expression of markers such as neurofilament, calbindin, synaptophysin or PLP. At later time points p53Myc and p53MA astrocytes became immunoreactive for Musashi-1, nestin, CD133 and Olig2 and negative for GFAP suggesting a SVZ type C cell-like differentiation. Prominin-1/CD133 is a five-domain transmembrane protein involved in cholesterol and iron metabolism that inhibitis clathrin-dependent endocytosis[55]. In the experiments described here, CD133 was localized in the cytoplasm of transduced astrocytes of later passages. Cytoplasmic localization has been observed in various human neoplasms and is correlated with an unfavourable prognosis in rectal cancer [56]. Immunoblotting of protein extracts from the transduced astrocytes described here revealed that the CD133 protein was a truncated form of 70kDa. This molecular weight has been described before in human tumor cells[29]. The biological significance of this protein fragment, however, remains obscure.

These results demonstrate that the expression of c-Myc and/or Akt is sufficient to induce several stem cell markers in cell lines derived from mature cortical astrocytes. A similar transformation of p53^−/−^ astrocytes has been shown for H-ras^V12^ [57]. Although stem cell markers can discriminate different subpopulations of tumor cells in GBM, the TIC remains an enigma. Cultured tumor cells are highly dynamic in the expression of stem cell markers and CD133 positive cells, widely regarded as glioma stem cells, can develop from initially CD133 negative cells after prolonged culturing [58]. Our results presented here, demonstrate that mature astrocytes can develop a morphologically undifferentiated phenotype resembling stem cells. Therefore, mature astrocytes should not be excluded from the pool of potential glioma initiating cells.

### Tumor model

In order to determine whether oncogene-transduced astrocytes had gliomagenic potential, we implanted p53MA astrocytes into the striate body of congenic mice. The tumorigenic potential of p53^−/−^ cells in the skin and the brain of nude mice has been previously reported [10], [11]. The comparable tumor size in mice with and without lymphocytes together with the lack of significant lymphocytic infiltration in the brains of C57BL/6 mice indicates that these tumors are well tolerated by the immune system. This finding contrasts to previously reported findings derived from other tumor models such as GL261 transplants that induce an immune response in the brain [59], [60]. These findings demonstrate that p53MA astrocytes represent a feasible model system for the generation of brain tumors. While the lack of lymphocytic infiltration resembled the observations in the human disease, we did not see diffuse infiltration of the surrounding brain by the implanted tumor cells. Transduction of the astrocytes with additional genes such as proteases might render these cells infiltrative into the surrounding brain.

Together, expression of c-Myc and Akt induces a progenitor-like phenotype of mature p53^−/−^ astrocytes and these cells are highly tumorigenic in the brain of immunocompetent mice. These cells might represent a valuable tool for the investigation of glioma development and biology.

## Supporting Information

Figure S1 **Morphological alterations after transduction for c-Myc and/or Akt.** Astrocytes of a 3^rd^ passage of C57BL/6 wt (A) and p53Ctrl (B) cell cultures revealed a normal, polygonal, astrocytic star-shape. Asterisk in B indicates a binucleate cell. Most third-passage p53AKT astrocytes (C) revealed an astrocyte-like shape. Many cells, however, showed an increased nuclear size and prominent nucleoli (inset). p53AKT astrocytes of a 12^th^ passage (F, I) showed an increased size of the cytoplasm and nuclei (arrows in I). A significantly decreased cell size was seen in early-passage p53MYC (D). Late-passage p53MYC astrocytes (G, K) revealed an increased nuclear-cytoplasmic ratio and short bipolar cell processes. Note the large number of apoptotic cells (asterisks). Early-(E) and late passage (H) p53MA cultures showed a highly heterogeneous picture with small, bipolar as well as very large multinucleated cells (inset in E, arrows in L). (Scale bar: A–E: 100 µm, F–H: 50 µm).
(TIF)
