# Correction: Akt and c-Myc Induce Stem-Cell Markers in Mature Primary p53^−/−^ Astrocytes and Render These Cells Gliomagenic in the Brain of Immunocompetent Mice

**DOI:** 10.1371/annotation/562d2594-464f-49f1-90ef-f78cafcd3956

**Published:** 2013-10-23

**Authors:** Josefine Radke, Ginette Bortolussi, Axel Pagenstecher

There is an error in the Legend of Figure 6. The correct legend is: "Injection of p53MA astrocytes of an 8th passage led to large tumors in two out of three mice (A, B) while p53MA astrocytes of a 3rd passage resulted in the development of clearly smaller tumors (arrows) of similar size in C57BL/6 (C) and Rag 2−/− (D) mice. Tumors in C57BL/6 mice revealed only few CD4+ (E, arrow)-, no CD8+ (F) - T-cells and no MAC-1 immunoreactive macrophages (G, scale bar: A–D: 500 µm, E–G: 100 µm)."

There is an additional error in the second to last sentence of the Results section under the subheading "Astrocytes expressing c-Myc and Akt are tumorigenic in the brain and are tolerated by the immune system." The correct sentence is: "Immunohistochemical stainings were performed to identify infiltrating T-cell subtypes and macrophages. In wild type C57BL/6 mice, there were only occasionally CD4+ T-cells and neither CD8+ T-cells nor MAC-1 immunoreactive macrophages were detected in the transplants (Figure 6 E-G). The similar size of tumors in wild type and Rag2−/− mice together with the absence of invading mononuclear cells in and around the transplants in wild type mice indicates that there was no significant immune response against p53MA astrocytes in the brain of congenic C57BL/6 mice." 

