# Supplementary figures and images for: Akt and c-Myc Induce Stem-Cell Markers in Mature Primary p53−/− Astrocytes and Render These Cells Gliomagenic in the Brain of Immunocompetent Mice
Source: PLoS One. 2013 Feb 12;8(2):e56691. doi: 10.1371/journal.pone.0056691 (PMC3570527; doi:10.1371/journal.pone.0056691)

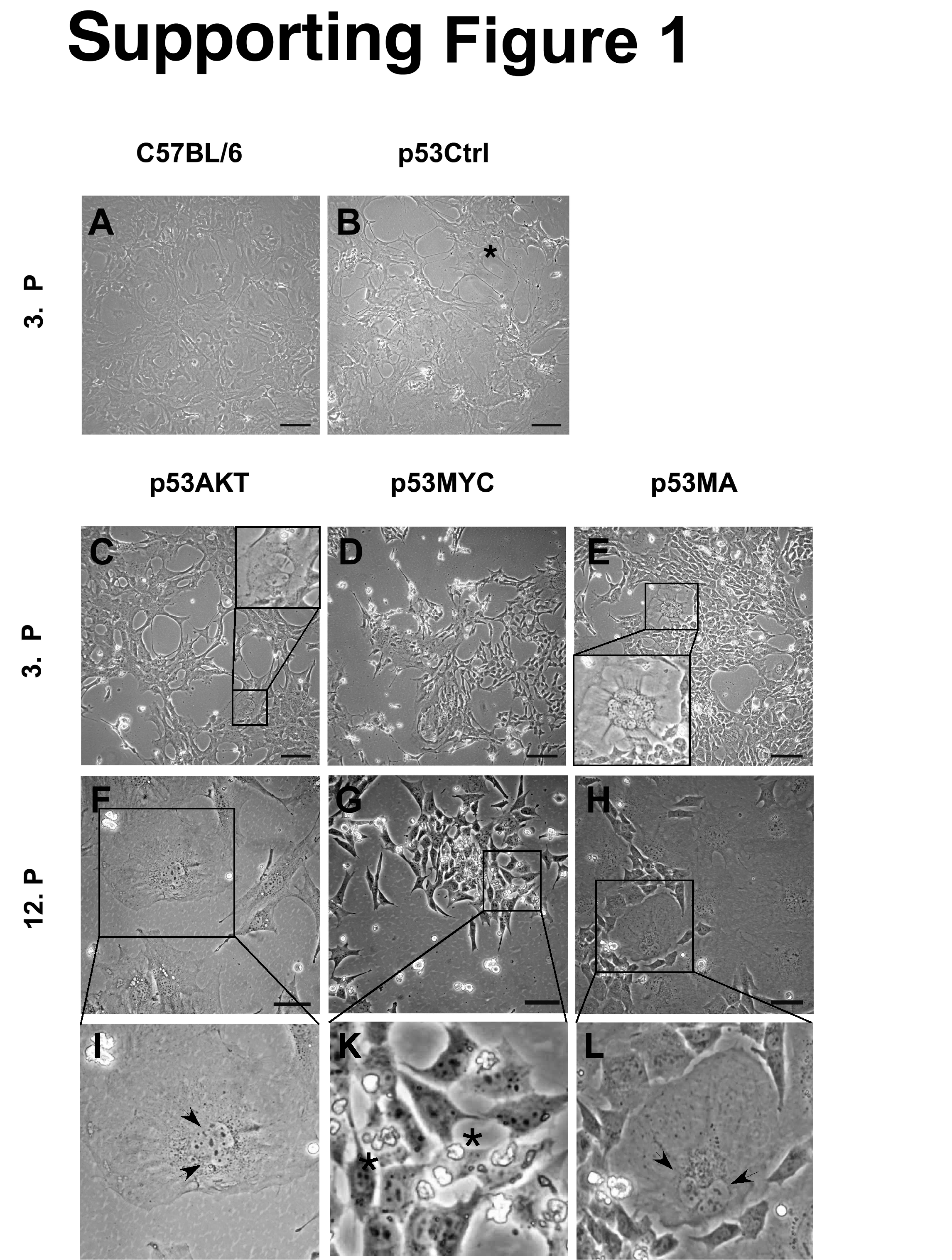

Supplement: Figure S1 — Morphological alterations after transduction for c-Myc and/or Akt. Astrocytes of a 3rd passage of C57BL/6 wt (A) and p53Ctrl (B) cell cultures revealed a normal, polygonal, astrocytic star-shape. Asterisk in B indicates a binucleate cell. Most third-passage p53AKT astrocytes (C) revealed an astrocyte-like shape. Many cells, however, showed an increased nuclear size and prominent nucleoli (inset). p53AKT astrocytes of a 12th passage (F, I) showed an increased size of the cytoplasm and nuclei (arrows in I). A significantly decreased cell size was seen in early-passage p53MYC (D). Late-passage p53MYC astrocytes (G, K) revealed an increased nuclear-cytoplasmic ratio and short bipolar cell processes. Note the large number of apoptotic cells (asterisks). Early-(E) and late passage (H) p53MA cultures showed a highly heterogeneous picture with small, bipolar as well as very large multinucleated cells (inset in E, arrows in L). (Scale bar: A–E: 100 µm, F–H: 50 µm). (TIF) [file pone.0056691.s001.tif]
